# *In vitro* effect of ferrous sulphate on bovine spermatozoa motility parameters, viability and Annexin V-labeled membrane changes

**DOI:** 10.1371/journal.pone.0257766

**Published:** 2021-09-23

**Authors:** Zuzana Knazicka, Hana Duranova, Veronika Fialkova, Michal Miskeje, Tomas Jambor, Alexander V. Makarevich, Shubhadeep Roychoudhury, Anton Kovacik, Peter Massanyi, Norbert Lukac

**Affiliations:** 1 Faculty of Biotechnology and Food Sciences, Slovak University of Agriculture, Nitra, Slovak Republic; 2 AgroBioTech Research Centre, Slovak University of Agriculture, Nitra, Slovak Republic; 3 BioFood Centre, Faculty of Biotechnology and Food Sciences, Slovak University of Agriculture, Nitra, Slovak Republic; 4 Research Institute for Animal Production in Nitra, National Agricultural and Food Centre, Luzianky, Slovak Republic; 5 Department of Life Science and Bioinformatics, Assam University, Silchar, India; 6 Department of Animal Physiology, Faculty of Biotechnology and Food Sciences, Slovak University of Agriculture, Nitra, Slovak Republic; Universite Clermont Auvergne, FRANCE

## Abstract

The aim of this study was to assess the dose- and time-dependent *in vitro* effects of ferrous sulphate (FeSO_4_.7H_2_O) on the motility parameters, viability, structural and functional activity of bovine spermatozoa. Spermatozoa motility parameters were determined after exposure to concentrations (3.90, 7.80, 15.60, 31.20, 62.50, 125, 250, 500 and 1000 μM) of FeSO_4_.7H_2_O using the SpermVision^TM^ CASA (Computer Assisted Semen Analyzer) system in different time periods. Cell viability was assessed by 3-(4,5-dimethylthiazol-2-yl)-2,5- diphenyltetrazolium bromide (MTT) assay, and the Annexin V-Fluos was applied to detect the membrane integrity of spermatozoa. The initial spermatozoa motility showed increased average values at all experimental concentrations compared to the control group (culture medium without FeSO_4_.7H_2_O). After 2 h, FeSO_4_.7H_2_O stimulated the overall percentage of spermatozoa motility at the concentrations of ≤ 125 μM. However, experimental administration of 250 μM of FeSO_4_.7H_2_O significantly (P < 0.001) decreased the spermatozoa motility but had no negative effect on the cell viability (P < 0.05) (Time 2 h). The lowest viability was noted after the addition of ≥ 500 μM of FeSO_4_.7H_2_O (P < 0.001). The concentrations of ≤ 62.50 μM of FeSO_4_.7H_2_O markedly stimulated (P < 0.001) spermatozoa activity after 24 h of exposure, while at high concentrations of ≥ 500 μM of FeSO_4_.7H_2_O the overall percentage of spermatozoa motility was significantly inhibited (P < 0.001) and it elicited cytotoxic action. Fluorescence analysis confirmed that spermatozoa incubated with higher concentrations (≥ 500 μM) of FeSO_4_.7H_2_O displayed apoptotic changes, as detected in head membrane (acrosomal part) and mitochondrial portion of spermatozoa. Moreover, the highest concentration and the longest time of exposure (1000 μM of FeSO_4_.7H_2_O; Time 6 h) induced even necrotic alterations to spermatozoa. These results suggest that high concentrations of FeSO_4_.7H_2_O are able to induce toxic effects on the structure and function of spermatozoa, while low concentrations may have the positive effect on the fertilization potential of spermatozoa.

## Introduction

The natural environmental factors and differentiated anthropogenic pollutants, as well as many other sources strongly influence the male reproductive system, both in animals and humans [1, 2]. Exposure to heavy metals is a risk factor in the assessment of spermatogenesis [3], while certain trace elements have been shown to be essential for testicular development and spermatogenesis [4], as well as for the preservation of the fertilization capacity of spermatozoa [5].

Based on the widespread use, key roles in biological processes and bilateral role in the organism, trace element iron (Fe) has been chosen for the present *in vitro* reprotoxicity study. In the human body, Fe has a crucial role as part of metalloproteins such as hemoglobin or myoglobin, as well as enzymes that are associated with energetic reactions [6]. Furthermore, it is a biologically essential element of every living organism because Fe cofactors activate enzymes involved in major metabolic processes in the cell. Indeed, Fe participates in various physiological, regulatory, and biochemical processes such as oxidation/reduction reactions, electron transport associated with cellular respiration, deoxyribonucleic acid (DNA) synthesis/repair, cell division and proliferation, all of which are closely related to spermatozoa production and metabolism [7–17]. In effect, it has been reported that Fe-containing enzymes play a key role in spermatogenesis and semen quality, because Sertoli and Leydig cells are important sources of Fe storage protein, ferritin [8, 18]. Recently, Chao et al. [19] have confirmed crosstalk between sex hormones and ferritin at the systemic level. Data from epidemiological studies suggest that the concentrations of Fe in the environment have increased due to anthropogenic activities including agricultural practices [20], biomass burning [21], steel industry, sewage, and dust from mining [22]. As a result of environmental pollution, Fe concentrations in the environment have been changing rapidly and its homeostasis in the cells may get disrupted as a result [23]. At physiological levels, Fe and its compounds have not been reported to be toxic for the animals and humans [2]. Nevertheless, disturbances in the regulative absorption mechanism can appear due to pathological conditions or prolonged intake of high Fe doses. In these cases, since the capacity for storage of Fe in ferritin is exceeded, the metal is complexed to phosphate or hydroxide to form hemosiderin (or it is bound into proteins), and in this form it is present in the liver [3]. Indeed, Fe excess in the organism is associated with the metal deposition in organs throughout the body (mainly liver, heart, and endocrine glands) [24, 25] and relates to their specific damages. Moreover, Fe can induce cell death by generating free radicals as it interconverts between ferrous (Fe^2+^) and ferric (Fe^3+^) forms [24].

Previously, the biometal has been reported to be required for the proper development and functioning of the male reproductive system [26]. However, it may become highly detrimental if accumulated in large quantities [27]. Excessive doses of Fe resulted in testicular atrophy with morphological changes and lesions in the seminiferous tubules, epididymis and Sertoli cells, impaired spermatogenesis, associated with pathological disorders and impaired reproductive performance [8, 10, 28–32]. Sperm DNA damage [33, 34] as well as toxic impact of long-term dietary Fe (300 ppm for 100 days) overload on gene expression and enzyme activity of the testicular antioxidant defence system [35] have also been reported. In mice, alterations in male reproduction (related to decreased epididymis-body weight ratio, increased seminal vesicle-body weight ratio along with severe deterioration of testicular microstructure) have been observed after short-term exposure to Fe (0.30 mg/day for 18 days) [36]. Other studies have showed the association between mean Fe concentration in the seminal plasma and spermatozoa progressive motility as well as viability [37]. Similarly, Tvrda et al. [18] quantified Fe in seminal plasma which was positively correlated with spermatozoa motility characteristics (P < 0.05). They also revealed that Fe is important for the preservation of spermatozoa motility and antioxidant power at physiological amounts only. Decreased levels of Fe were found in males diagnosed with asthenozoospermia [38]. On the other hand, intravenous Fe application in men with Fe deficiency anemia caused a doubling of spermatozoa count and improvement all the semen parameters [39].

Spermatozoa are extremely sensitive to various factors which may disturb the process of spermatogenesis and consequently lead to a decrease in spermatozoa quality and production. Iron has indispensable roles in the physiology, as well as pathology of male reproduction. Published studies highlight the crucial roles of Fe in cellular respiration, spermatozoa development and metabolism [13]. To our knowledge, there is little information available on the impact of Fe (in the form of ferrous sulphate–FeSO_4_.7H_2_O) on the fertilization potential of the spermatozoa. Hence, this *in vitro* study was carried out to obtain more insight in reference to the concentrations used in the few previous studies [14, 18, 40, 41]. The present study aimed to investigate the dose- and time-dependent effects of FeSO_4_.7H_2_O on the motility parameters, viability as well as structural and functional activity of bovine spermatozoa.

## Materials and methods

### Biological material

Bovine semen samples (n = 58) were obtained from 6 adult Holstein-Friesian breeding bulls and processed following routine methods at the bull breeding station (Slovak Biological Services, Nitra, Slovak Republic). The selected bulls were from 1 to 6 years old, and the frequency of semen collection was once weekly (early in the morning). Each ejaculate was obtained from each bull on regular collection schedule using an artificial vagina. The samples had to accomplish the basic quality criteria given for the corresponding breed. After processing, the samples were stored in the laboratory at room temperature (22–25°C) and basic measurements were performed–volume (mL), pH, concentration (x10^9^/mL) and osmolarity (mOsmol/kg) according to standard methods [42]. The results of basic semen parameters showed that all observed characteristics were within the physiological range (Table 1). Each sample was diluted in physiological saline solution (sodium chloride 0.90% w/v; Bieffe Medital, Grosotto, Italy; pH 6.50–7.00, osmolarity 301–308 mOsmol/kg), using a dilution ratio of 1:40.

**Table 1:** The basic parameters of bovine semen samples (n = 58).

PARAMETERS	x ± SD
ph	6.56 ± 0.20
Spermatozoa concentration (x10^9^/mL)	3.15 ± 0.96
Semen volume (mL)	6.23 ± 1.69
Osmolarity (mOsmol/kg)	297.50 ± 4.67
Spermatozoa Fe concentration (μg/mL)	0.049*
Seminal plasma Fe concentration (μg/mL)	0.025**

x–arithmetic mean, ± SD–standard deviation

* The Fe contents in spermatozoa were determined by the flame atomic absorption spectrophotometry (FAAS).

** The seminal plasma Fe concentrations were analyzed by UV/VIS spectrophotometry.

### Samples processing

The samples were centrifuged (10 min, 9500 rpm, 4 ˚C) to obtain the cell sediment (spermatozoa) and seminal plasma fraction. The fractions were separated and transferred into 1.50 mL tubes and kept frozen (-80˚C).

### Assessment of spermatozoa Fe concentration

Spermatozoa (cell sediment) were mineralized by adding 1.0 mL of HNO_3_ (65%; Sigma-Aldrich, St. Louis, MO, USA). The resulting solution was diluted to 3.0 mL with demineralized water. Concentrations of Fe in spermatozoa were determined by direct aspiration of the acidic sample into the flame atomic absorption spectrophotometry (FAAS). This complies with the specification for standardized FAAS quick procedure for metals when using the BUCK Model 200A atomic absorption spectrophotometer (Cole-Parmer International, Court Vernon Hills, Illinois, USA). The quantification limit for Fe was 0.12 mg/L and the detection limit 0.0036 mg/L. Calibration Fe was delineated using suitable standard concentrations (0.125, 0.25, 0.50, 1.0 and 10 μg/g) by diluting standard (0.50% HNO_3_). Concentrations were expressed as μg/mL.

### Assessment of seminal plasma Fe concentration

The analysis of seminal plasma Fe concentration was determined using the BioLa Test commercial kit (PLIVA-Lachema, Brno, Czech Republic) according to the manufacturer’s instructions. In a pH 4.80 buffer system, Fe is released from transferrin and then quantitatively reduced to ferrous state. Fe^2+^ forms with Ferene S [(3-(2-pyridyl)-5,6-bis-2-(5-furylsulfonic acid)-1,2,3-triazine)] a stable, coloured complex, whose colour intensity is proportional to the amount of Fe in the sample. The interference from copper is eliminated by particular reaction conditions and a specific masking agent. The absorbance was measured at 593 nm using the Genesys 10 spectrophotometer (Thermo Fisher Scientific Inc., Madison, USA). Concentrations were expressed as μg/mL.

### *In vitro* exposure

The metal, in the form of FeSO_4_.7H_2_O (≥ 99%; Sigma-Aldrich, St. Louis, USA) was dissolved directly in physiological saline solution and added to the semen samples at final concentrations of 3.90, 7.80, 15.60, 31.20, 62.50, 125, 250, 500 and 1000 μM. Subsequently, pH and osmolarity of the culture medium were checked after FeSO_4_.7H_2_O addition within the entire range of concentrations (pH = 5.70–6.40; osmolarity 298–310 mOsmol/kg in case 3.90–1000 μM of FeSO_4_.7H_2_O). The spermatozoa with FeSO_4_.7H_2_O were cultured in 96-well plates (MTP, Greiner, Germany) (at 37°C). The experimental groups A-I (exposed to the respective concentrations of FeSO_4_.7H_2_O as mentioned above) were compared with the control group (Ctrl—culture medium without FeSO_4_.7H_2_O).

### Spermatozoa motility analysis

The spermatozoa motility was analyzed using the Computer Assisted Semen Analyzer (CASA) system–SpermVision^TM^ program (MiniTȕb, Tiefenbach, Germany) with the Olympus BX 51 phase-contrast microscope (Olympus, Tokyo, Japan) equipped with heating plate (37°C). Each sample was placed into a Makler Counting Chamber with a depth of 10 μm (Sefi-Medical Instruments, Haifa, Israel) and using the bovine specific set the following parameters were evaluated: percentage of motile spermatozoa (%; motility *>* 5 μm/s), percentage of progressively motile spermatozoa (%; motility *>* 20 μm/s), velocity average path (VAP; μm/s) and distance average path (DAP; μm) in different time periods (Times 0 h, 1 h, 2 h and 24 h). In each sample at least 1500–1700 spermatozoa were analyzed. Results of the analysis were collected of ten repeated experiments at each concentration.

### Cytotoxicity evaluation

Viability of the cells exposed to FeSO_4_.7H_2_O was evaluated by MTT [3-(4,5- dimethylthiazol-2-yl)-2,5-diphenyltetrazolium bromide] assay [43], a standard colorimetric assay which measures the conversion of a yellow water-soluble tetrazolium salt to purple formazan particles by mitochondrial succinate dehydrogenase enzyme (mitochondrial reductase) of living cells. The formazan was measured spectrophotometrically. In brief, the cultured 3.15x10^9^ cells/mL (in 200 μL medium per well) in 96-well plates (MTP, Greiner, Germany) were stained with MTT reagent (Sigma-Aldrich, St. Louis, MO, USA) which was dissolved in Dulbecco’s Phosphate Buffer Saline (Sigma, St. Louis, USA) at 5 mg/mL and added to the cells (in 20 μL per well). After incubation at Times 0 h, 1 h, 2 h and 24 h (at 37°C), the cells and the formazan crystals were dissolved in 80 μL of isopropanol (2-propanol, p.a. CentralChem, Bratislava, Slovak Republic). The absorbance was determined at a measuring wavelength of 570 nm against 620 nm as reference by a microplate ELISA reader (Multiscan FC, ThermoFisher Scientific, Finland). The data were expressed in percentage of the control group (i.e., absorbance of formazan from cells not exposed to FeSO_4_.7H_2_O) [44]. Results of the analysis were collected during seven repeated experiments at each concentration (n = 23–32).

### Fluorescence analysis

Membrane phosphatidylserine (PS) translocation (membrane destabilization) was detected after staining with fluorescently labeled Annexin V using an Annexin V-Fluos staining kit (Roche Diagnostics GmbH, Mannheim, Germany). Detection of spermatozoa with disordered membrane was carried out for selected FeSO_4_.7H_2_O concentrations representing the range in different time periods (Times 0 h, 2 h and 6 h). For the Annexin V analysis semen samples were washed in a binding HEPES-buffered saline (supplied with a kit) and centrifuged (2000 rpm for 6 min at 4°C; Universal 320/320R, Tuttlingen, Germany). The semen suspension (5.0 μL) was mixed with 100 μL working solution of Annexin V-Fluos and incubated for 20 min at 37°C. Afterwards, an aliquot of the semen suspension was placed between the microslide and coverslip in 5.0 μL of the anti-fade medium Vectashield containing DAPI fluorescent dye (4’,6-diamidino-2-phenylindol; blue image, cell DNA) and propidium iodide fluorescent dye (PI, red fluorescence, necrosis) according to the manufacturer’s instructions with slight modifications. The staining with Annexin V was immediately checked under a Leica DMR 270 fluorescent microscope (Leica Microsystems, Wetzlar, Germany) at 400x magnification using a 488 nm wavelength filter. The spermatozoa with disordered membrane (PS asymmetry) exhibited green fluorescence, whilst live spermatozoa (with intact membrane) remained unstained [45–47]. Use of a combination of two fluorescent dyes, Annexin V and PI, led to the identification of three distinct types of spermatozoa: (i) viable spermatozoa (Annexin V-negative, PI-negative, and DAPI-positive); (ii) dead spermatozoa (Annexin V-positive, PI-positive, DAPI-positive); and (iii) spermatozoa with impaired but integer plasma membrane (Annexin V-positive, PI-negative and DAPI-positive). Experiments were repeated five times. In each experiment, about 8–10 microscopic fields were viewed per group and photographed. In total, more than 1500 spermatozoa were counted in each experimental group.

### Statistical analysis

Obtained data were statistically analyzed using the PC program GraphPad Prism 3.02 (GraphPad Software Incorporated, San Diego, California, USA). Descriptive statistical characteristics (arithmetic mean, minimum, maximum, standard deviation, and coefficient of variance) were evaluated. Homogeneity of variance was assessed by Bartlett’s test. One-way analysis of variance (ANOVA) and the Dunnett’s multiple comparison tests were used for statistical evaluations. The level of significance was set at ^***^ (P *<* 0.001); ^**^ (P *<* 0.01); ^*^ (P *<* 0.05).

## Results

### Concentration of Fe in semen

The quantified Fe in seminal plasma was 0.025 μg/mL and spermatozoa Fe concentration measured by the FAAS method was 0.049 μg/mL. Results of the analyses are presented in Table 1.

### Ferrous sulphate and spermatozoa motility parameters

**Fig 1:**
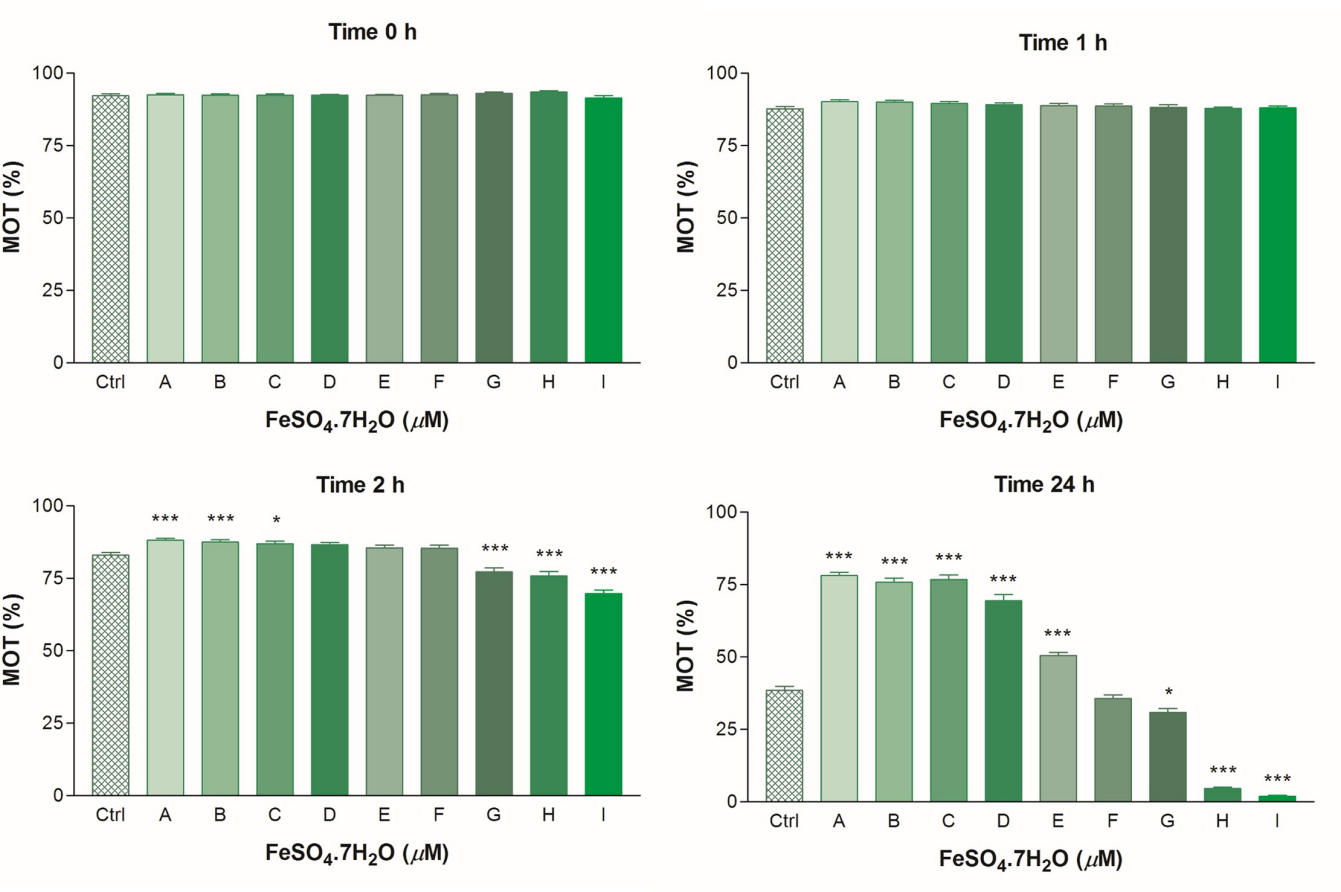
Spermatozoa motility (MOT; %) exposed to FeSO_4_.7H_2_O in different time periods. The control group (Ctrl) received a culture medium without FeSO_4_.7H_2_O administration; Group A– 3.90 μM; B– 7.80 μM; C– 15.60 μM; D– 31.20 μM; E– 62.50 μM; F– 125 μM; G– 250 μM; H– 500 μM; I– 1000 μM of FeSO_4_.7H_2_O. Results of the analysis were obtained of 10 repeated experiments at each concentration. The statistical difference between the values of Ctrl and treated spermatozoa was indicated by asterisks ***P < 0.001; **P < 0.01; *P < 0.05 (One-way ANOVA with Dunnett’s multiple comparison test). CASA system.

Results of the effect of *in vitro* supplementation of FeSO_4_.7H_2_O on bovine spermatozoa motility are presented in Fig 1. Initially (at Times 0 h and 1 h), similar values for the percentages of motile spermatozoa were noted in all the groups. After 2 h, the average motility values significantly (P *<* 0.001) decreased at the concentrations ≥ 250 μM of FeSO_4_.7H_2_O in comparison with the control group. However, a significant increase of spermatozoa motility at the concentrations of 15.60 μM (P < 0.05) and ≤ 7.80 μM (P < 0.001) of FeSO_4_.7H_2_O was recorded. Iron at the concentrations of ≤ 62.50 μM of FeSO_4_.7H_2_O after long-term (Time 24 h) supplementation markedly stimulated (P < 0.001) the spermatozoa motility. The lowest motility of spermatozoa was significantly detected in the groups with high concentrations of FeSO_4_.7H_2_O (P < 0.05 in case 250 μM; P < 0.001 in case ≥ 500 μM) (Time 24 h). Identical spermatozoa motility was also detected for the percentage of progressively motile spermatozoa during all the time periods (Table 2).

**Table 2:** Progressive spermatozoa motility (%) exposed to FeSO_4_.7H_2_O in different time periods.

Groups	Control	3.90	7.80	15.60	31.20	62.50	125	250	500	1000
	Ctrl	A	B	C	D	E	F	G	H	I
FeSO_4_.7H_2_O (μM)										
**Time 0 h**										
x	88.68	90.31	90.56	90.43	90.19	89.49	89.30	89.25	91.18^**C**^	88.94
minimum	70.58	79.48	81.25	76.47	79.48	79.41	74.57	72.41	80.51	77.08
maximum	94.93	97.72	95.91	98.71	97.56	96.77	96.59	96.73	97.60	97.00
± SD	5.42	4.71	3.74	5.16	4.21	4.54	5.62	5.95	3.89	5.91
CV (%)	6.11	5.22	4.12	5.70	4.66	5.07	6.29	6.67	4.27	6.64
**Time 1 h**										
x	82.90	86.52^**C**^	86.12	85.87	85.40	85.09	85.52	84.50	83.36	83.54
minimum	60.00	72.13	69.56	67.07	57.14	69.44	67.89	62.16	53.22	60.46
maximum	93.18	97.05	98.37	98.55	96.84	96.52	94.73	97.18	93.68	92.85
± SD	7.79	6.23	6.91	7.10	7.04	7.76	6.59	8.41	7.20	7.50
CV (%)	9.39	7.20	8.02	8.27	8.25	9.12	7.70	9.95	8.64	8.97
**Time 2 h**										
x	78.24	85.18^**A**^	84.55^**A**^	81.76	81.35	80.10	79.58	72.99	69.46	65.45
minimum	65.20	59.18	54.54	50.74	56.47	61.19	54.76	55.55	46.34	52.27
maximum	89.65	94.36	97.10	96.62	91.66	94.82	95.83	88.40	80.88	80.43
± SD	7.08	7.36	8.01	11.19	8.29	8.31	11.01	9.40	10.19	7.62
CV (%)	9.05	8.64	9.47	13.69	10.19	10.38	13.84	12.89	14.68	11.65
**Time 24 h**										
x	34.01	72.06^**A**^	71.00^**A**^	70.97^**A**^	64.19^**A**^	44.16^**A**^	30.86	24.11^**C**^	2.25^**A**^	1.16^**A**^
minimum	25.20	53.19	47.45	41.79	51.44	31.25	20.90	19.17	1.03	1.00
maximum	47.91	86.11	88.07	86.79	75.12	63.46	42.85	29.41	3.43	1.21
± SD	6.60	9.20	12.78	13.53	8.12	9.03	7.41	3.39	0.78	0.09
CV (%)	19.41	12.77	18.00	19.06	12.65	20.45	24.00	14.06	34.46	7.75

x–arithmetic mean, ± SD–standard deviation, CV (%)–coefficient of variation

^A^P < 0.001

^B^P < 0.01

^C^P < 0.05.

Detailed data describing the spermatozoa motility parameters–VAP (μm/s) and DAP (μm) are shown in Tables 3 and 4. The VAP analysis revealed significant (P < 0.001) differences at the concentrations of ≤ 15.60 μM of FeSO_4_.7H_2_O in comparison with the control group after Time 1 and 2 h. The highest concentrations (≥ 500 μM) of FeSO_4_.7H_2_O significantly (P < 0.001) decreased the VAP (Time 2 h). After Time 24 h, the spermatozoa exposed to low concentrations (≤ 62.50 μM) of FeSO_4_.7H_2_O (P < 0.001) became more active than those in the control group, but their exposure to higher concentrations (≥ 500 μM) of FeSO_4_.7H_2_O resulted in a significant (P < 0.001) decreased. Evaluation of DAP showed similar results as to the velocity parameters. The DAP analysis revealed significant differences at the concentrations of ≤ 15.60 μM of FeSO_4_.7H_2_O in comparison to the control group (Times 1 h and 2 h). Interestingly, the concentration of 125 μM of FeSO_4_.7H_2_O at short-term supplementation stimulated the spermatozoa motility, but gradually (after Time 24 h) inhibited the selected parameter. Other data were significant (P < 0.001) in comparison with the control group, and a time- as well as dose-dependent tendency was noted, too.

**Table 3:** Velocity average path of spermatozoa (μm/s) exposed to FeSO_4_.7H_2_O in different time periods.

Groups	Control	3.90	7.80	15.60	31.20	62.50	125	250	500	1000
	Ctrl	A	B	C	D	E	F	G	H	I
FeSO_4_.7H_2_O (μM)										
**Time 0 h**										
x	91.02	92.38	92.17	91.95	91.84	91.10	91.51	91.41	90.50	90.34
minimum	79.74	81.16	81.36	81.25	77.10	79.90	79.61	80.61	76.97	77.33
maximum	103.90	109.90	109.80	106.50	110.90	110.10	108.20	105.80	109.30	102.70
± SD	7.47	5.79	6.55	5.82	8.40	8.09	8.25	7.89	9.29	7.10
CV (%)	8.21	6.27	7.11	6.33	9.15	8.88	9.02	8.63	10.26	7.86
**Time 1 h**										
x	81.86	86.36^**A**^	86.34^**A**^	86.93^**A**^	84.37	82.13	82.85	82.18	78.68	76.60
minimum	68.12	64.29	70.18	68.84	69.06	71.85	64.27	61.22	61.58	65.22
maximum	94.61	109.60	103.70	103.60	102.50	98.81	99.17	96.88	99.56	93.56
± SD	6.72	10.15	8.42	7.55	7.83	6.56	8.03	6.89	10.76	7.95
CV (%)	8.20	11.76	9.75	8.69	9.28	7.98	9.69	8.38	13.67	10.37
**Time 2 h**										
x	75.43	85.30^**A**^	84.45^**A**^	81.42^**A**^	77.90	76.99	76.92	71.37	65.38^**A**^	56.33^**A**^
minimum	60.04	58.23	66.44	62.74	57.05	62.07	56.75	51.75	43.22	41.23
maximum	92.74	99.37	99.72	99.19	96.31	100.20	103.30	80.80	82.87	73.51
± SD	8.49	10.16	9.14	8.66	10.39	9.98	10.57	7.29	11.46	9.52
CV (%)	11.25	11.92	10.82	10.63	13.34	12.97	13.74	10.22	17.54	16.91
**Time 24 h**										
x	35.90	66.33^**A**^	63.94^**A**^	60.86^**A**^	54.06^**A**^	45.06^**A**^	31.39	30.55	5.07^**A**^	2.23^**A**^
minimum	26.42	47.67	44.32	40.11	32.19	32.79	22.67	18.54	3.22	0.00
maximum	48.54	86.96	87.62	79.84	69.59	64.83	40.70	46.89	7.97	3.65
± SD	4.27	15.88	16.47	12.17	14.04	9.58	4.08	8.14	2.05	1.08
CV (%)	11.88	23.94	25.75	20.00	25.97	21.26	12.99	26.64	40.33	48.53

x–arithmetic mean, ± SD–standard deviation, CV (%)–coefficient of variation

^A^P < 0.001

^B^P < 0.01

^C^P < 0.05.

**Table 4:** Distance average path of spermatozoa (μm) exposed to FeSO_4_.7H_2_O in different time periods.

Groups	Control	3.90	7.80	15.60	31.20	62.50	125	250	500	1000
	Ctrl	A	B	C	D	E	F	G	H	I
FeSO_4_.7H_2_O (μM)										
**Time 0 h**										
x	37.10	38.80^**C**^	38.21^**C**^	39.05^**C**^	38.92^**C**^	37.40	37.29	37.70	36.17	35.62
minimum	31.22	33.02	34.38	32.56	32.53	33.49	32.00	32.03	29.60	27.69
maximum	43.33	45.62	48.52	44.88	47.00	43.50	46.00	47.00	45.40	42.97
± SD	3.50	2.90	3.12	2.82	3.69	2.58	3.66	4.29	4.71	4.24
CV (%)	9.45	7.47	8.18	7.21	9.49	6.89	9.82	11.39	13.01	11.90
**Time 1 h**										
x	35.80	37.37^**C**^	37.10^**C**^	37.20^**C**^	36.23	35.43	35.22	35.11	32.66	31.34
minimum	27.98	30.09	31.37	28.70	30.86	31.02	30.02	30.09	27.59	27.93
maximum	41.29	44.92	47.08	46.30	43.61	42.77	43.82	42.48	41.05	35.56
± SD	3.01	3.49	3.32	3.47	3.22	2.90	2.93	2.97	3.72	1.92
CV (%)	8.40	9.33	8.95	9.34	8.87	8.19	8.32	8.46	11.38	6.12
**Time 2 h**										
x	33.09	36.18^**A**^	35.94^**A**^	35.34^**C**^	34.02	33.73	33.63	31.11^**C**^	29.14^**A**^	25.98^**A**^
minimum	20.05	23.87	28.38	28.09	23.07	21.22	22.88	21.86	20.93	20.09
maximum	40.38	40.61	42.04	42.99	43.79	44.32	47.91	39.52	36.69	33.96
± SD	4.10	3.82	3.52	3.47	5.24	4.93	6.46	5.09	4.66	3.90
CV (%)	12.38	10.56	9.79	9.81	15.39	14.61	19.19	16.36	15.99	15.00
**Time 24 h**										
x	16.53	25.23^**A**^	25.25^**A**^	24.12^**A**^	22.90^**A**^	16.57^**A**^	12.07^**A**^	10.92^**A**^	1.67^**A**^	0.00^**A**^
minimum	12.21	18.41	15.34	14.55	15.05	13.84	8.27	6.84	1.01	0.00
maximum	19.47	38.72	37.63	34.91	30.96	19.67	14.94	14.32	2.85	0.00
± SD	1.59	6.70	6.95	5.52	6.07	1.46	1.84	1.95	0.67	0.00
CV (%)	9.64	26.56	27.53	22.90	26.49	8.82	15.21	17.81	40.26	0.00

x–arithmetic mean, ± SD–standard deviation, CV (%)–coefficient of variation

^A^P < 0.001

^B^P < 0.01

^C^P < 0.05.

### Ferrous sulphate and viability of spermatozoa

**Fig 2:**
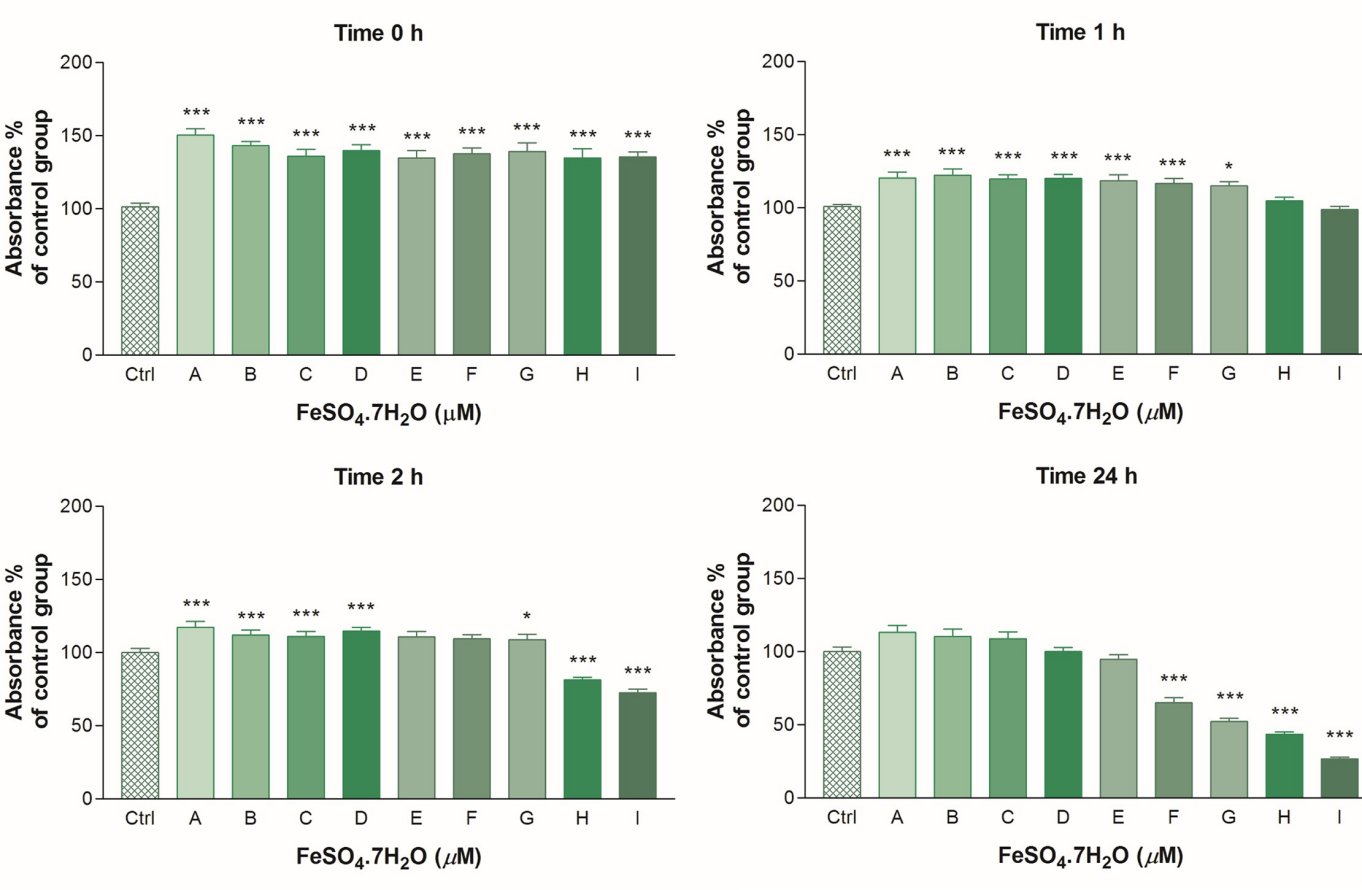
The effect of FeSO_4_.7H_2_O on the viability of spermatozoa in different time periods. Each bar represents the arithmetic mean (± SD) absorbance in % of (untreated) control group (Ctrl), which represented 100%. Results of the analysis were obtained of 7 independent experiments at each concentration. A decline in absorbance reflects a decline in the cell viability. The control group (Ctrl) received a culture medium without FeSO_4_.7H_2_O administration; Group A– 3.90 μM; B– 7.80 μM; C– 15.60 μM; D– 31.20 μM; E– 62.50 μM; F– 125 μM; G– 250 μM; H– 500 μM; I– 1000 μM of FeSO_4_.7H_2_O. The statistical difference between the values of Ctrl and treated spermatozoa was indicated by asterisks ***P < 0.001; **P < 0.01; *P < 0.05 (One-way ANOVA with Dunnett’s multiple comparison test). MTT assay.

The viability of spermatozoa (Fig 2) as detected by the MTT assay was significantly (P < 0.001) higher in all the experimental groups (Time 0 h) in comparison to the control group. After Time 1 h, a significant (P < 0.05) increase of cell viability was noted at the concentrations of ≤ 250 μM (P < 0.001 in case ≤ 125 μM) of FeSO_4_.7H_2_O in comparison with the control group. The lowest viability (P < 0.001) was determined after the addition of ≥ 500 μM of FeSO_4_.7H_2_O (Time 2 h). In addition, a concentration of 250 μM of FeSO_4_.7H_2_O during short-term supplementation significantly (P < 0.001) decreased the spermatozoa motility parameters but had no apparent negative impact on the mitochondrial activity of spermatozoa (P < 0.05). After Time 24 h, the survival of the cells decreased proportional to the increasing concentrations of FeSO_4_.7H_2_O. At lower concentrations, FeSO_4_.7H_2_O was able to maintain cell viability after long-term supplementation; however, this increase was not significant statistically (P > 0.05). The lowest viability of spermatozoa (P < 0.001) was detected at the concentrations of ≥ 125 μM of FeSO_4_.7H_2_O.

### Ferrous sulphate and Annexin V-labeled structural changes in spermatozoa

**Fig 3:**
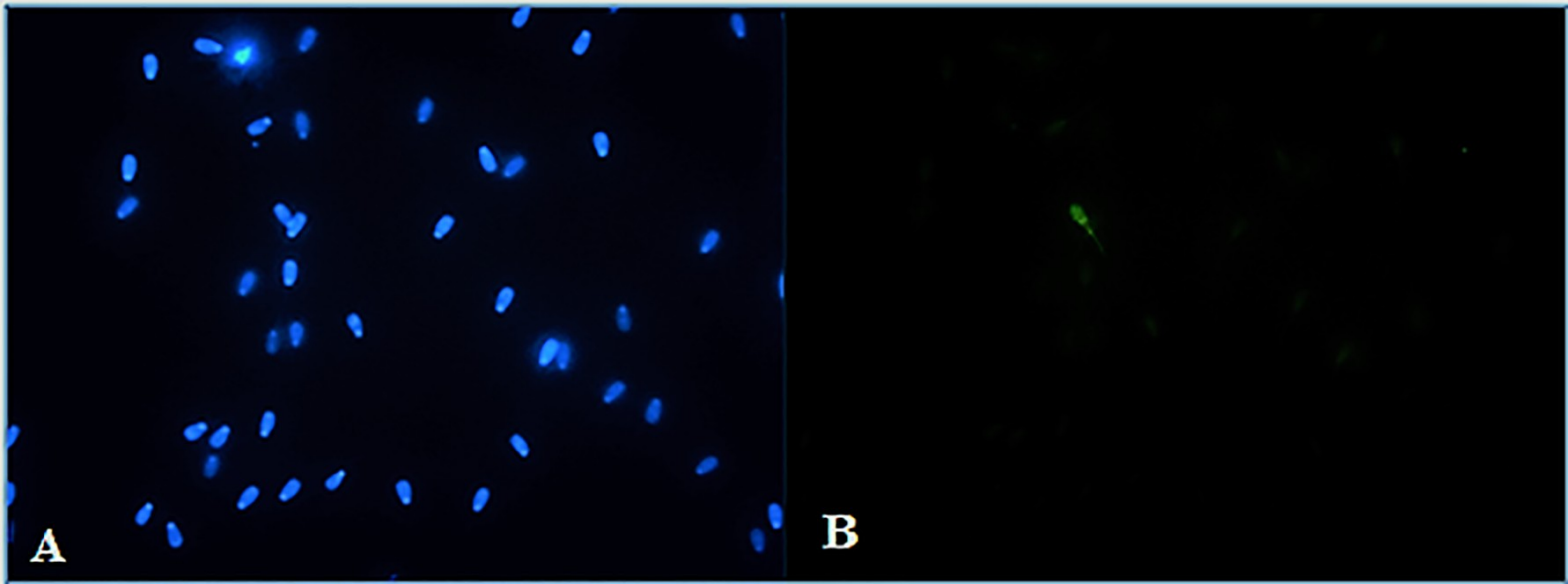
Fluorescent staining of bovine spermatozoa in the control group without FeSO_4_.7H_2_O administration after Time 2 h of culture. Blue-stained (DAPI positive) chromatin of spermatozoa heads (A). The cells are Annexin V-negative (B), without apoptotic changes (400x magnification).

**Fig 4:**
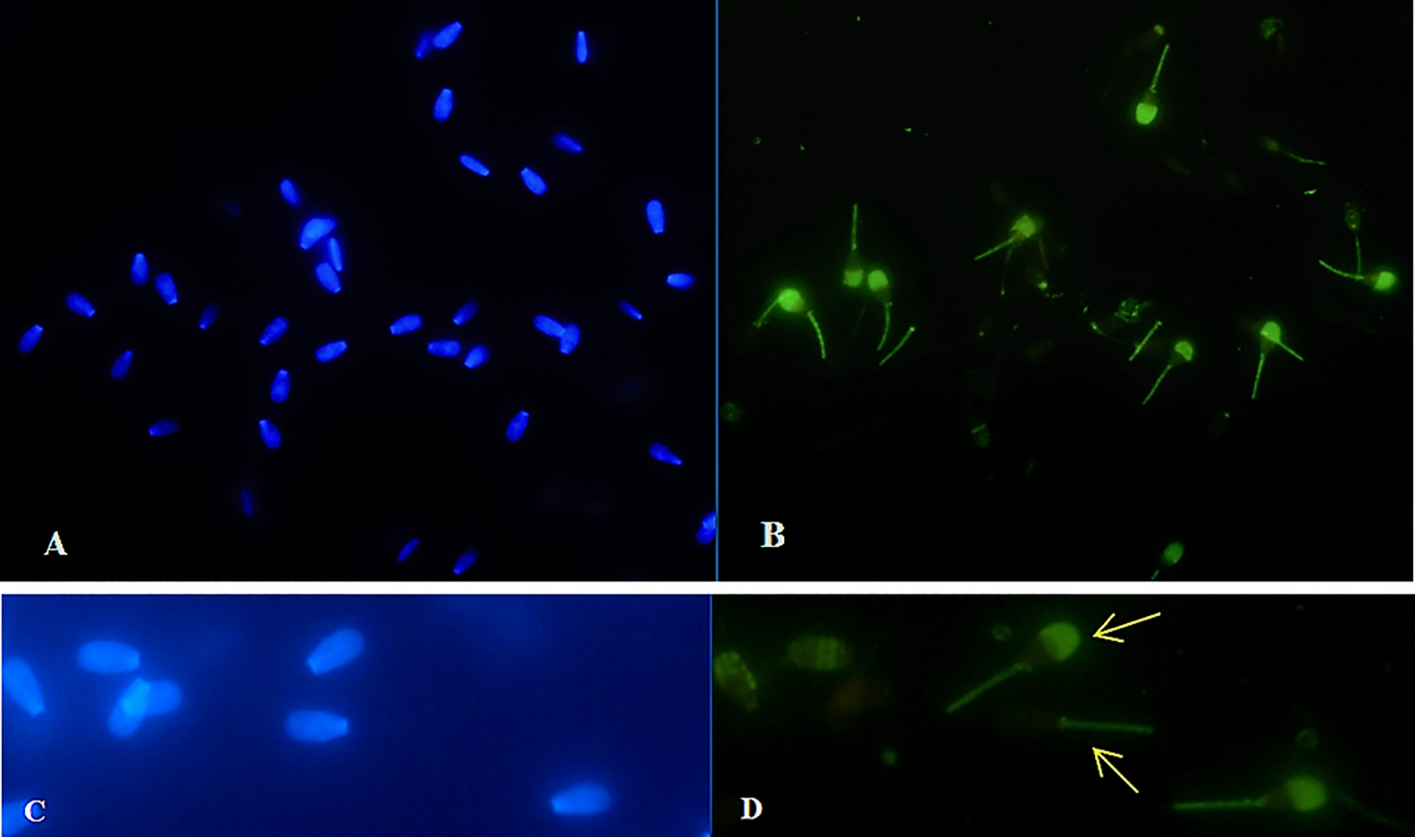
*In vitro* culture of spermatozoa with 500 μM of FeSO_4_.7H_2_O after Time 6 h. Blue-stained (DAPI positive) chromatin of spermatozoa heads (A, C). Detection of apoptosis in spermatozoa (green staining) (B). Annexin V fluorescence reaction was detected in the mitochondrial segment and head (acrosomal part) of bovine spermatozoa (D; arrow) (400x magnification).

**Fig 5:**
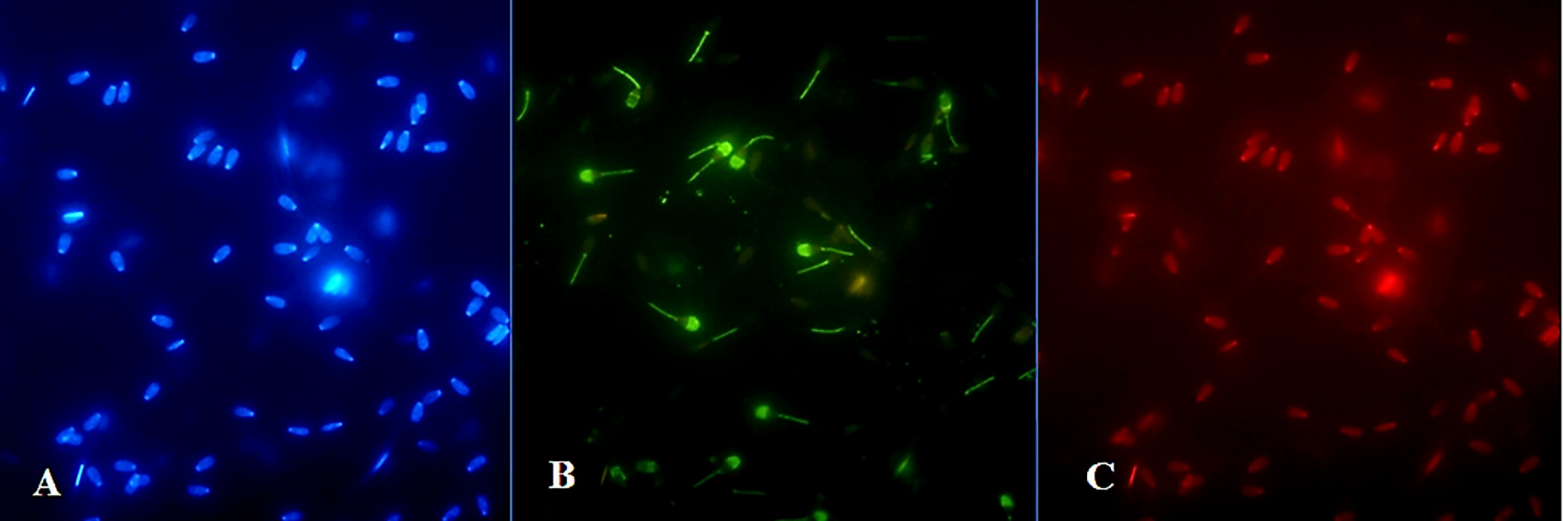
*In vitro* culture of spermatozoa with the highest concentration (1000 μM) of FeSO_4_.7H_2_O after Time 6 h. Blue-stained (DAPI positive) chromatin of spermatozoa heads (A). Typical apoptotic (B) as well as necrotic spermatozoa alterations (C), fluorescently detected by propidium iodide (red staining) (400x magnification).

**Fig 6:**
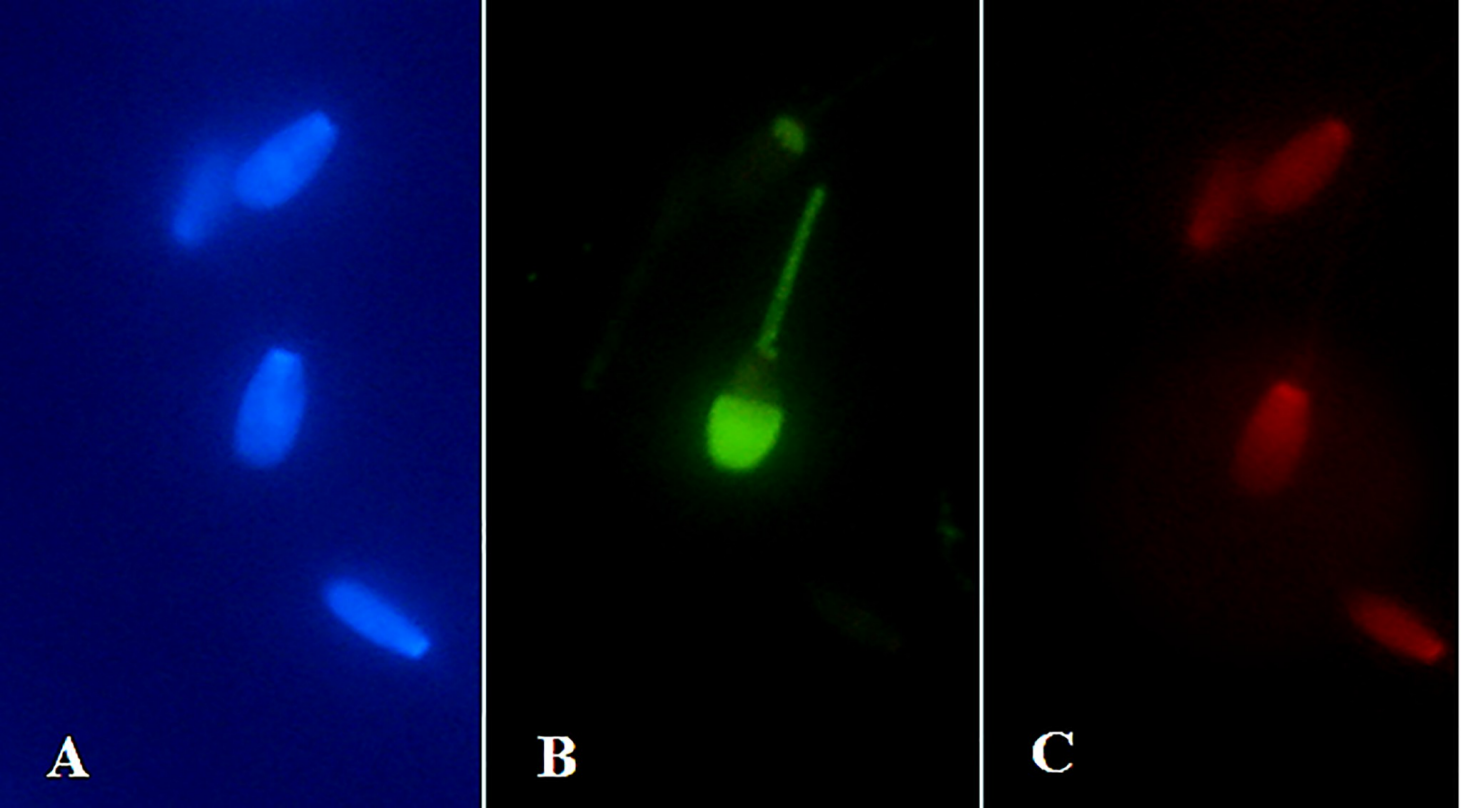
More detailed image of altered spermatozoa in the group with the highest concentration (1000 μM) of FeSO_4_.7H_2_O and the longest time of exposure (6 h). Blue-stained (DAPI positive) chromatin of spermatozoa heads (A). Annexin-positive regions of apoptotic changes (B) were found not only in the mitochondrial portion, but also on the spermatozoa head (acrosomal part). Necrosis positivity by propidium iodide at the excitation 488 nm was detected (C) (400x magnification).

Results from fluorescence analysis are presented in Figs 3–6. Detection of spermatozoa with disordered membrane was carried out for the groups with higher concentrations (≥ 500 μM) of FeSO_4_.7H_2_O. The highest concentration (1000 μM) of FeSO_4_.7H_2_O after Time 2 h caused typical apoptotic process. *In vitro* supplementation of spermatozoa with 500 μM of FeSO_4_.7H_2_O after Time 6 h was detected by the Annexin V fluorescence reaction on the mitochondrial portion and head (acrosomal part) of bovine spermatozoa. In the group with the highest concentration and the longest time of exposure (1000 μM of FeSO_4_.7H_2_O; Time 6 h), characteristic Annexin-positive regions in the mitochondrial segment and in the spermatozoa head membrane (acrosomal part) were also detected, showing a significant alteration of spermatozoa membrane integrity. In addition, this concentration induced even necrotic spermatozoa alterations (fluorescently detected by PI).

## Discussion

Iron is an essential trace nutrient playing important roles in spermatogenesis [2]. Like other risk elements, depending on its level in the organisms, Fe is capable of demonstrating its dual (essential and toxic) actions. Therefore, the target of our *in vitro* study was to analyze the dose- and time-dependent effects of FeSO_4_.7H_2_O on the spermatozoa and to evaluate its relationship with spermatozoa quality parameters including motility, viability, and functional as well as structural integrity. The results of the present CASA analysis revealed that the concentrations of ≤ 125 μM of FeSO_4_.7H_2_O stimulated the overall spermatozoa motility during short-term culture. It may be postulated that reproductive cells exhibit an increased demand for intracellular Fe, because the metal has a pivotal role in cellular homeostasis as a substrate or cofactor of enzymes. Beneficial impact of Fe (≤ 62.50 μM; P < 0.001) on the spermatozoa motility parameters was observed after long-term culture, too. This may be explained by the unique position of Fe among other biometals, and the transferrin cycle and regulation of Fe homeostasis acts to keep the amount of free ferrous Fe at the lowest possible level. On the other hand, the high concentrations of ≥ 500 μM of FeSO_4_.7H_2_O (P < 0.05 in case 250 μM of FeSO_4_.7H_2_O) induced a toxic effect, resulting in a notable decrease of the spermatozoa activity. The gradual decrease of spermatozoa motility in the experimental groups supplemented with high Fe doses may be acknowledged to the oxidative stress to which spermatozoa are subjected during the *in vitro* culture. These results may be considered as a follow-up of our previous study [40], which detailed the assessment of a wide range of concentrations of FeSO_4_.7H_2_O on the spermatozoa motility parameters *in vitro*. Significant increase of spermatozoa motility parameters in relation to FeSO_4_.7H_2_O addition was reported. Based on these findings, it may be concluded that FeSO_4_.7H_2_O at low concentrations maintains the spermatozoa motility parameters, but at high concentrations acts as a toxic agent. Our results also point out that Fe in acceptable concentrations probably has a direct action on the fertilization potential of spermatozoa. Similarly, Tvrda et al. [41] experimentally confirmed that Fe^2+^ or Fe^3+^ a dose- and time-dependent impact on spermatozoa. The dual roles of Fe on spermatozoa motility were noted at Time 16 h. High concentrations of both forms are toxic, while concentrations below 10 μmol/L of FeCl_2_ together with 50 μmol/L of FeCl_3_ proved to exert favourable effects on spermatozoa motility (P < 0.001). In accordance with our results, the lowest motility was recorded in both experimental groups with the highest concentrations (1000 μmol/L) of FeCl_2_ and FeCl_3_ (Time 24 h), resulting in a notable decrease of the spermatozoa activity, accompanied by oxidative balance. We consider that high concentrations of transition metal ions such as Fe cations can disrupt the mechanism of activity of the spermatozoa. Comparing our results with those obtained in a previous work by Tvrda et al. [41], wherein chlorides were used, it seems that rather high concentrations of Fe cations than sulphate anions have adverse effects on the spermatozoa. Probably the kind of the salt of Fe is not as important factor for toxicological evaluation.

Iron is necessary component of bovine semen and is needed for a proper spermatozoa function. Currently, there is also a considerable lack of data referring concentration of Fe in spermatozoa or only in seminal plasma fraction. Despite the complex relationship between semen analysis and trace elements, we quantified Fe in the whole semen, which is an important factor for determining the fertilization potential of spermatozoa. The present study is the first, which completely evaluates total concentration of Fe in bovine semen and reflects its presence in the spermatozoa (0.049 μg/mL) versus seminal plasma (0.025 μg/mL). Our observation showed higher concentration of Fe in the cell sediment (spermatozoa), which seems representative of cumulative exposures of this trace element.

Importance of Fe in male fertility has been shown in several *in vivo* and *in vitro* studies. Kodama et al. [26] reported that incubation of spermatozoa with low concentrations of Fe significantly improves fertilization rates of mice. Syamsunarno et al. [36] observed that the mice spermatozoa motility increases in dose-dependent manner (although not significant) after short-term Fe overload injection. Hashemi et al. [48] studied relationships between the seminal plasma levels of trace elements and evaluated their effects on human spermatozoa motility parameters. The increased Fe levels caused a decrease in most of spermatozoa motility fractions. Huang et al. [49] observed that incubation of spermatozoa with Fe^2+^ caused a reduction of motility associated with marked lipid peroxidation. Although excessive doses of Fe cause destructive effect on the testicular function and spermatogenesis [29], its physiological level is necessary for optimal spermatozoa function [50]. Aydemir et al. [51] showed a positive correlation between serum levels of Fe and spermatozoa motility. Positive correlation between spermatozoa motility and lactotransferrin levels in seminal plasma from ejaculates have been found in the study by Kiso et al. [52]. Differences in Fe plasma seminal levels have also been reported in subfertile subjects [53, 54]. Kanwal et al. [8] found significant positive correlation between Fe contents in semen and motile spermatozoa percentage of crossbred cow bulls. These observations are supported by Eghbali et al. [37], too.

Spermatozoa motility is closely associated with mitochondria which are helically arranged around the axoneme in the sperm mid-piece and play a key role in energy production crucial for the sperm movement [55–58]. In the present study, the viability of spermatozoa after exposure to FeSO_4_.7H_2_O was assessed by the MTT assay. The formazan particles were reduced by the mitochondrial enzyme succinate dehydrogenase [43], which selectively accumulate in living cells. Ruiz-Pesini et al. [59] clearly demonstrated a direct and positive correlation between spermatozoa motility and mitochondrial respiratory chain enzyme activities. It is known that motility is closely related to the functional activity of the sperm mitochondria, as adenosine triphosphate (ATP) interconnects the motion activity of the spermatozoon with the functional stability of its energetic centre [58]. Results of the present study indicate a significant (P < 0.001) increase in spermatozoa viability at low concentrations (≤ 125) μM of FeSO_4_.7H_2_O which is in agreement with enhanced motility of spermatozoa. These concentrations supported the mitochondrial activity of spermatozoa (P < 0.001). Mitochondrial ATP synthesis using the ATPase complex transports energy into the cells [57], which is required for a wide range of spermatozoa functions, particularly for the motility [60]. In the present study, high concentrations of ≥ 500 μM of FeSO_4_.7H_2_O, however, significantly (P < 0.001) reduced the spermatozoa motility and also elicited cytotoxic effect. It appears that these concentrations decreased the mitochondrial activity of spermatozoa or enzymatic complex, and subsequently the spermatozoa were not able to utilize this energy (ATP). The reduced percentage of motility with a low viability of spermatozoa may reflect structural abnormalities and/or metabolic alterations of the cells, too. Interestingly, the concentration of 250 μM of FeSO_4_.7H_2_O in short-term culture demonstrably inhibited (P < 0.001) the spermatozoa motility parameters but had no negative effect on the mitochondrial activity of spermatozoa (P < 0.05). This observation points to another possible mechanism of its toxicity which could be reflected via other cellular pathways. A deeper understanding of mitochondrial energy metabolism could open up new avenues in the investigation of Fe action on spermatozoa mitochondrial bioenergetics, both in physiological and pathological conditions.

The mitochondria are the primary source of reactive oxygen species (ROS), small amounts of which are necessary for the spermatozoon to acquire the fertilizing capacity [61]. On the other hand, the organelle is a major site of intracellular ROS production within most mammalian cells which underlies mitochondrial oxidative damage in many pathological processes and contributes to retrograde redox signalling from the mitochondria to cytosol and the nucleus [62]. Mitochondria are a target of toxic actions of many substances [63] which may amplify their oxidative damage. Iron as transition metal possesses this ability and thus, it may be preferentially toxic to cells with high mitochondrial activity [64]. Bauckman et al. [65] found that ovarian carcinoma cell lines treated with 250 μmol/L of non-transferrin bound Fe during 24 h induced mitochondrial damage, reduced expression of outer mitochondrial membrane proteins, increased ROS levels and reduced cell viability. Indeed, several researchers have suggested that the cytotoxic effects of metals (including trace elements such as Fe) are dependent upon the chemical form, valence states (inorganic versus organic), length of exposure, time-duration, route of administration, various experimental models, as well as the doses used and apart from many other factors [40, 66, 67].

Lucesoli et al. [68] reported that disproportionate levels of Fe^2+^ can reduce the size of testes. Smaller testes and reduced sperm production may be related to the elevated Fe^2+^ concentrations [29]. Severe Fe overload increases oxidative stress in testes and epididymal sperm causing infertility [69]. Wise et al. [10] showed that the Fe concentration was negatively correlated with the transferrin or ferritin availability and testicular weight in boars. Furthermore, boars with high Fe levels and low transferrin/ferritin produced less sperm. As the testicular Fe concentration increased, daily sperm production and total daily sperm production declined. The study concluded that abnormal activity of both transferrin and ferritin were associated with hypogonadism and Fe accumulation may lead to reduced sperm production. According to Massanyi et al. [53, 70], increased Fe concentration directly affects the spermatozoa morphology. The present study revealed that structural and functional alterations of spermatozoa are associated with Fe toxicity. Furthermore, fluorescence analysis confirmed that spermatozoa incubated with high concentrations (500 and 1000 μM) of FeSO_4_.7H_2_O displayed apoptotic changes, mainly detected in the head membrane (acrosomal part) and mitochondrial portion of spermatozoa. Moreover, the highest concentration and the longest time of exposure (1000 μM of FeSO_4_.7H_2_O; Time 6 h) induced even necrotic spermatozoa alterations. During the early phases of disturbed membrane function, asymmetry of membrane phospholipids occurs prior to a progressively disturbed integrity of the cytoplasmic membrane. The disturbance of membrane function starts with the translocation of PS from the inner to the outer leaflet of the plasma membrane and results in an exposure of PS on the external surface. This translocation of PS is one of the earliest features of cells undergoing apoptosis. Annexin V staining enables the identification of cells with deteriorating membrane integrity at an earlier stage than staining with supravital stains [45, 71]. The detection of PS exposure, as a well-established early marker of sperm apoptosis has been applied in various studies [72–74]. Gandini et al. [75] examined the morphological aspect of the apoptotic spermatozoa. Based on the results of the present study, it may be assumed that the apoptotic alterations in the spermatozoa may be associated with various forms of abnormal spermatozoa morphology resulting in reduced motility and viability of spermatozoa. In this regard, Ammar et al. [76] provided clear evidence that the apoptotic alterations are closely correlated with the morphological features of spermatozoa, especially to the head and the tail shape. The defiance or overload of seminal trace elements or enzymes may cause functional and qualitative defects on spermatozoon. Positive correlations were found between increased levels of Fe and apoptotic sperm markers. The authors also supported the hypothesis that increased Fe level may be an important factor involved in the mechanism of oxidative stress-mediated apoptosis in teratozoospermic semen. Generally, the deleterious effects of Fe as a transition metal are attributed to its ability to generate ROS. According to Agarwal and Saleh [77], the toxicity of Fe results from the Fenton and Haber-Weiss reactions, resulting in the formation of highly toxic hydroxyl free radicals from hydrogen peroxide and superoxide ion radicals, which can affect lipids, proteins, and the nuclear DNA [27, 78].

## Conclusions

The results of the present *in vitro* study revealed the dose- and time-dependent effects of FeSO_4_.7H_2_O on spermatozoa parameters including motility, viability, and functional structural integrity. Beneficial impact of FeSO_4_.7H_2_O (≤ 62.50 μM) on bovine spermatozoa motility parameters was observed after long-term culture (Time 24 h). Low concentrations of FeSO_4_.7H_2_O showed a favourable effect on cell viability and thus, on the energy metabolism, which is a key factor supporting spermatozoa activity. On the other hand, high concentrations (≥ 500 μM) of FeSO_4_.7H_2_O are able to induce a toxic (and cytotoxic) effect, resulting in a notable decrease of the spermatozoa motility parameters and viability, accompanied by structural alterations (head membrane and mitochondrial portion of spermatozoa). In functional aspects, all these changes could disrupt the mechanism of motion activity of the spermatozoon. We consider that the adverse effects of higher concentrations of FeSO_4_.7H_2_O on the spermatozoa are caused by the effect of Fe cations. Furthermore, results of the present study point out that Fe in acceptable concentrations probably has a direct action on the fertilization potential of the spermatozoa, which could be used in assisted reproductive technologies.

## References

[1] Fergusson JE. The Heavy Elements: Chemistry, Environmental Impact and Health Effects. Oxford: Pergamon Press; 1990.

[2] Marzec-Wróblewska U, Kamiński P, Łakota P. Influence of chemical elements on mammalian spermatozoa. Folia Biol. 2012; 58: 7–15. 22464819 10.14712/fb2012058010007

[3] Kabata-Pendias A, Mukherjee AB. Trace Elements from Soil to Human, 1. ed., Germany Heidelberg: Springer; 2007.

[4] Eidi M, Eidi A, Pouyan O, Shahmohammadi P, Fazaeli R, Bahar M. Seminal plasma levels of copper and its relationship with seminal parameters. Iran J Reprod Med. 2010; 8: 60–65.

[5] Atig F, Raffa M, Ben-Ali H, Kerkeni A, Saad A, Mounir A. Impact of seminal trace element and glutathione levels on semen quality of Tunisian infertile men. BMC Urology. 2012; 12: 6–14. doi: 10.1186/1471-2490-12-6 22429816 PMC3349502

[6] Dev S, Babitt JL. Overview of iron metabolism in health and disease. Hemodial Int. 2017; 1: 6–20. doi: 10.1111/hdi.12542 28296010 PMC5977983

[7] Carter ED. Oxidation-reduction reactions of metal ions. Environ Health Perspect. 1995; 103: 17–19. doi: 10.1289/ehp.95103s117 7621791 PMC1519346

[8] Kanwal MR, Rehman NU, Ahmad N, Samad HA, Zia-Ur-Rehman HA, Akhtar N, et al. Bulk cations and trace elements in the Nili-ravi buffalo and crossbred cow bull semen. Int J Agri Biol. 2000; 2: 203–205.

[9] Lieu PT, Heiskala M, Peterson PA, Yang Y. The roles of iron in health and disease. Mol Aspects Med. 2001; 22: 1–87. doi: 10.1016/s0098-2997(00)00006-6 11207374

[10] Wise T, Lunstra DD, Rohrer GA, Ford JJ. Relationships of testicular iron and ferritin concentrations with testicular weight and sperm production in boars. J Anim Sci. 2003; 81: 503–511. doi: 10.2527/2003.812503x 12643495

[11] Wang J, Pantopoulos K. Regulation of cellular iron metabolism. Biochem J. 2011; 434: 365–381. doi: 10.1042/BJ20101825 21348856 PMC3048577

[12] Abbaspour N, Hurrell R, Kelishadi R. Review on iron and its importance for human health. J Res Med Sci. 2014; 19:164–174. 24778671 PMC3999603

[13] Tvrda E, Peer R, Sikka SC, Agarwal A. Iron and copper in male reproduction: a double-edged sword. J Assist Reprod Genet. 2015; 32: 3–16. doi: 10.1007/s10815-014-0344-7 25245929 PMC4294866

[14] Tvrda E, Lukac N, Lukacova J, Jambor T, Massanyi P. Dose- and time-dependent *in vitro* effects of divalent and trivalent iron on the activity of bovine spermatozoa. Biol Trace Elem Res. 2015; 167: 36–47. doi: 10.1007/s12011-015-0288-5 25758720

[15] Gozzelino R, Arosio P. Iron homeostasis in health and disease. Int J Mol Sci. 2016; 17: 130. doi: 10.3390/ijms17010130 26805813 PMC4730371

[16] Keller MA, Zylstra A, Castro C, Turchyn AV, Griffin JL, Ralser M. Conditional iron and pH-dependent activity of a non-enzymatic glycolysis and pentose phosphate pathway. Sci Adv. 2016; 2: e1501235. doi: 10.1126/sciadv.1501235 26824074 PMC4730858

[17] Puig S, Ramos-Alonso L, Romero AM, Martinez-Pastor MT. The elemental role of iron in DNA synthesis and repair. Metallomics. 2017; 9: 1483–1500. doi: 10.1039/c7mt00116a 28879348

[18] Tvrda E, Knazicka Z, Lukacova J, Schneidgenova M, Massanyi P, Goc Z, et al. Relationships between iron and copper content, motility characteristics, and antioxidant status in bovine seminal plasma. J Microbiol Biotechnol Food Sci. 2012; 2: 536–547.

[19] Chao KC, Chang CC, Chiou HY, Chang JS. Serum ferritin is inversely correlated with testosterone in boys and young male adolescents: A cross-sectional study in Taiwan. PLoS One. 2015; 10: e0144238. doi: 10.1371/journal.pone.0144238 26646112 PMC4672881

[20] Prospero JM, Ginoux P, Torres O, Nicholson SE, Gill TE. Environmental characterization of global sources of atmospheric soil dust identified with the Nimbus 7 Total Ozone Mapping Spectrometer (TOMS) absorbing aerosol product. Rev Geophys. 2002; 40: 1–31.

[21] Fu HB, Shang GF, Lin J, Hu YJ, Hu QQ, Guo L, et al. Fractional iron solubility of aerosol particles enhanced by biomass burning and ship emission in Shanghai, East China. Sci Total Environ. 2014; 481: 377–391. doi: 10.1016/j.scitotenv.2014.01.118 24607631

[22] Reimann C, de Caritat P. Chemical Elements in the Environment. Berlin: Springer Verlag; 1998.

[23] Schreinemachers DM, Ghio AJ. Effects of environmental pollutants on cellular iron homeostasis and ultimate links to human disease. Environ Health Insights. 2016; 10: 35–43. doi: 10.4137/EHI.S36225 26966372 PMC4782969

[24] Iancu TC. Ultrastructural aspects of iron storage, transport and metabolism. J Neural Transm. 2011; 118: 329–335. doi: 10.1007/s00702-011-0588-7 21318635

[25] Eid R, Arab NTT, Greenwood MT. Iron mediated toxicity and programmed cell death: A review and a re-examination of existing paradigms. BBA–Molecular Cell Research. 2017; 1864: 399–430. doi: 10.1016/j.bbamcr.2016.12.002 27939167

[26] Kodama H, Kuribayashi Y, Gagnon C. Effect of sperm lipid peroxidation on fertilization. J Androl. 1996; 17: 151–157. 8723439

[27] Aitken RJ, Harkiss D, Buckingham DW. Relationship between iron-catalysed lipid peroxidation potential and human sperm function. J Reprod Fertil. 1993; 98: 257–265. doi: 10.1530/jrf.0.0980257 8345470

[28] Lucesoli F, Fraga CG. Oxidative damage to lipids and DNA concurrent with decrease of antioxidants in rat testes after acute iron intoxication. Arch Biochem Biophys. 1995; 316: 567–571. doi: 10.1006/abbi.1995.1076 7840668

[29] Merker HJ, Baumgartner W, Kovac G, Bartko P, Rosival I, Zezula I. Iron-induced injury of rat testis. Andrologia. 1996; 28: 267–273. doi: 10.1111/j.1439-0272.1996.tb02795.x 8893095

[30] De Pereira ML, e Costa FG. Spermatogenesis recovery in the mouse after iron injury. Hum Exp Toxicol. 2003; 22: 275–279. doi: 10.1191/0960327103ht344oa 12774891

[31] Carriquiriborde P, Handy RD, Davies SJ. Physiological modulation of iron metabolism in rainbow trout (*Oncorhynchus mykiss*) fed low and high iron diet. J Exp Biol. 2004; 207: 75–86. doi: 10.1242/jeb.00712 14638835

[32] Gunel-Ozcan A, Basar MM, Kisa U, Ankarali HC. Hereditary haemochromatosis gene (HFE) H63D mutation shows an association with abnormal sperm motility. Mol Biol Rep. 2009; 36: 1709–1714. doi: 10.1007/s11033-008-9372-7 18846434

[33] Perera D, Pizzey A, Campbell A, Katz M, Poter J, Petrou M, et al. Sperm DNA damage in potentially fertile homozygous ß-thalassaemia patients with iron overload. Hum Reprod. 2002; 17: 1820–1825. doi: 10.1093/humrep/17.7.1820 12093845

[34] Anderson D, Schmid TE, Baumgartner A. Male-mediated developmental toxicity. Asian J Androl. 2014; 16: 81–88. doi: 10.4103/1008-682X.122342 24369136 PMC3901885

[35] Kocpinar EF, Gonul Baltaci G, Ceylan H, Kalin SN, Erdogan O, Budak H. Effect of a prolonged dietary iron intake on the gene expression and activity of the testicular antioxidant defense system in rats. Biol Trace Elem Res. 2020; 195: 135–141. doi: 10.1007/s12011-019-01817-0 31309445

[36] Syamsunarno MRAA, Widyastuti R, Herlambang H, Lubis A, Ghozali M, Panigoro R. Short term iron overload injection alters reproduction organ and sperm quality in male mice. Adv Anim Vet Sci. 2021; 9: 35–41.

[37] Eghbali M, Alavi-Shoushtari SM, Asri-Rezai S, Ansari MHK. Effects of the seminal plasma iron and lead content on semen quality of water buffalo (*Bubalus bubalis*) bulls. Vet Res Forum. 2010; 1: 142–148.

[38] Skandhan KP, Mazumdar BN, Sumangala B. Study into the iron content of seminal plasma in normal and infertile subjects. Urologia. 2012; 79: 54–57. doi: 10.5301/RU.2012.9023 22328413

[39] Soliman A, Yassin M, De Sanctis V. Intravenous iron replacement therapy in eugonadal males with iron-deficiency anemia: Effects on pituitary gonadal axis and sperm parameters; A pilot study. Indian J Endocrinol Metab. 2014; 18: 310–316. doi: 10.4103/2230-8210.131158 24944924 PMC4056128

[40] Knazicka Z, Lukacova J, Tvrda E, Gren A, Goc Z, Massanyi P, et al. *In vitro* assessment of iron effect on the spermatozoa motility parameters. J Microbiol Biotechnol Food Sci. 2012; 2: 414–425.

[41] Tvrda E, Kovacik A, Tusimova E, Massanyi P, Lukac N. Changes in the antioxidant capacity and iron-binding properties of bovine spermatozoa following *in vitro* incubation with ferrous or ferric iron. Scientific Papers: Animal Science and Biotechnologies. 2015; 48: 31–39.

[42] Gamcik P, Kozumplik J, Mesaros P, Schvarc F, Vlcek Z, Zibrin M. Andrológia a inseminácia hospodárskych zvierat–Andrology and artificial insemination of farm animals (in Slovak). Bratislava: Publ. House Príroda; 1992.

[43] Mosmann T. Rapid colorimetric assay for cellular growth and surrival: application to proliferation and cytotoxicity assays. J Immunol Methods. 1983; 65: 55–63. doi: 10.1016/0022-1759(83)90303-4 6606682

[44] Knazicka Z, Tvrda E, Bardos L, Lukac N. Dose- and time-dependent effect of copper ions on the viability of bull spermatozoa in different media. J Environ Sci Health, Part A. 2012; 47: 1294–1300. doi: 10.1080/10934529.2012.672135 22540654

[45] Vermes I, Haanen C, Steffens-Nakken H. A novel assay for apoptosis. Flow cytometric detection of phosphatidylserine expression on early apoptotic cells using fluorescein labelled Annexin V. J Immunol Meth. 1995; 184: 39–51. doi: 10.1016/0022-1759(95)00072-i 7622868

[46] Makarevich AV, Chrenek P, Olexikova L, Popelkova M, Turanova Z, Ostro A, et al. Post-thaw survival, cell death and actin cytoskeleton in gene-microinjected rabbit embryos after vitrification. Theriogenology. 2008; 70: 675–681. doi: 10.1016/j.theriogenology.2008.04.043 18539321

[47] Lukac N, Bardos L, Stawarz R, Roychoudhury S, Makarevich AV, Chrenek P, et al. *In vitro* effect of nickel on bovine spermatozoa motility and Annexin V-labeled membrane changes. J Appl Toxicol. 2011; 31: 144–149. doi: 10.1002/jat.1574 20737413

[48] Hashemi MM, Behnampour N, Nejabat M, Tabandeh A, Ghazi-Moghaddam B, Joshaghani HR. Impact of seminal plasma trace elements on human sperm motility parameters. Rom J Intern Med. 2018; 56: 15–20. doi: 10.1515/rjim-2017-0034 28865234

[49] Huang YL, Tseng WC, Lin TH. *In vitro* effects of metal ions (Fe2+, Mn2+, Pb2+) on sperm motility and lipid peroxidation in human semen. J Toxicol Environ Health. 2001; 62: 259–267. doi: 10.1080/009841001459414 11245395

[50] Naes SM, Basri O, Ismail F, Ata’Allah GA, Idris SK, Mat Adenan NA, et al. Impact of elemental iron on human spermatozoa and mouse embryonic development in a defined synthetic culture medium. Reprod Biol. 2017; 17: 199–209. doi: 10.1016/j.repbio.2017.05.002 28532595

[51] Aydemir B, Kiziler AR, Onaran I, Alici B, Ozkara H, Akyolcu MC. Impact of Cu and Fe concentrations on oxidative damage in male infertility. Biol Trace Elem Res. 2006; 112: 193–203. doi: 10.1385/BTER:112:3:193 17057258

[52] Kiso WK, Selvaraj V, Nagashima J, Asano A, Brown JL, Schmitt DL, et al. Lactotransferrin in Asian elephant (*Elephas maximus*) seminal plasma correlates with semen quality. PLoS One. 2013; 8: e71033. doi: 10.1371/journal.pone.0071033 23976974 PMC3745378

[53] Massanyi P, Trandzik J, Nad P, Korenekova B, Skalicka M, Toman R, et al. Concentration of copper, iron, zinc, cadmium, lead, and nickel in bull and ram semen and relation to the occurrence of pathological spermatozoa. J Environ Sci Health, Part A. 2004; 39: 3005–3014. 15533020

[54] Marzec-Wróblewska U, Kamiński P, Łakota P, Szymański M, Wasilow K, Ludwikowski G, et al. Zinc and iron concentration and SOD activity in human semen and seminal plasma. Biol Trace Elem Res. 2011; 143: 167–177. doi: 10.1007/s12011-010-8868-x 20924714

[55] Jansen RPS, Burton GJ. Mitochondrial dysfunction in reproduction. Mitochondrion. 2004; 4: 577–600. doi: 10.1016/j.mito.2004.07.038 16120416

[56] Aziz DM. Assessment of bovine sperm viability by MTT reduction assay. Anim Reprod Sci. 2006; 92: 1–8. doi: 10.1016/j.anireprosci.2005.05.029 16023310

[57] Piomboni P, Focarelli R, Stendardi A, Ferramosca A, Zara V. The role of mitochondria in energy production for human sperm motility. Int J Androl. 2012; 35: 109–124. doi: 10.1111/j.1365-2605.2011.01218.x 21950496

[58] Duranova H, Valkova V, Knazicka Z, Olexikova L, Vasicek J. Mitochondria: A worthwhile object for ultrastructural qualitative characterization and quantification of cells at physiological and pathophysiological states using conventional transmission electron microscopy. Acta Histochem. 2020; 122: 151646. doi: 10.1016/j.acthis.2020.151646 33128989

[59] Ruiz-Pesini E, Diez C, Lapeña AC, Pérez-Martos A, Montoya J, Alvarez E, et al. Correlation of sperm motility with mitochondrial enzymatic activities. Clin Chem. 1998; 44: 1616–1620. 9702947

[60] Miki K. Energy metabolism and sperm function. Soc Reprod Fertil. 2007; Suppl 65: 309–325. 17644971

[61] Du Plessis SS, Agarwal A, Halabi J, Tvrda E. Contemporary evidence on the physiological role of reactive oxygen species in human sperm function. J Assist Reprod Genet. 2015; 32: 509–520. doi: 10.1007/s10815-014-0425-7 25646893 PMC4380893

[62] Murphy MP. How mitochondria produce reactive oxygen species. Biochem J. 2009; 417: 1–13. doi: 10.1042/BJ20081386 19061483 PMC2605959

[63] Meyer JN, Hartman JH, Mello DF. Mitochondrial toxicity. Toxicol Sci. 2018; 162: 15–23. doi: 10.1093/toxsci/kfy008 29340618 PMC5837373

[64] Eaton JW, Qian M. Molecular bases of cellular iron toxicity. Free Radic Biol Med. 2002; 32: 833–840. doi: 10.1016/s0891-5849(02)00772-4 11978485

[65] Bauckman K, Haller E, Taran N, Rockfield S, Ruiz-Rivera A, Nanjundan M. Iron alters cell survival in a mitochondria-dependent pathway in ovarian cancer cells. Biochem J. 2015; 466: 401–413. doi: 10.1042/BJ20140878 25697096 PMC4338747

[66] Mathur N, Pandey G, Jain GC. Male reproductive toxicity of some selected metals: A review. J Biol Sci. 2010; 10: 396–404.

[67] Calderon J, Ortiz-Pérez D, Yáňez L, Díaz-Barriga F. Human exposure to metals. Pathways of exposure, biomarkers of effect, and host factors. Ecotoxicol Environ Saf. 2003; 56: 93–103. doi: 10.1016/s0147-6513(03)00053-8 12915143

[68] Lucesoli F, Caligiuri M, Roberti MF, Perazzo JC, Fraga CC. Dose dependent increase of oxidative damage in the testes of rats subjected in acute iron overload. Arch Biochem Biophys. 1999; 372: 37–43. doi: 10.1006/abbi.1999.1476 10562414

[69] Huang YL, Tseng WC, Cheng SY, Lin TH. Trace elements and lipid peroxidation in human seminal plasma. Biol Trace Elem Res. 2000; 76: 207–215. doi: 10.1385/BTER:76:3:207 11049219

[70] Massanyi P, Trandzik J, Nad P, Skalicka M, Korenekova B, Lukac N, et al. Seminal concentration of trace elements in fox and relationships to spermatozoa quality. J Environ Sci Health, Part A. 2005; 40:1097–1105. doi: 10.1081/ese-200056166 15887577

[71] Glander HJ, Schaller J. Binding of annexin V to plasma membranes of human spermatozoa: rapid assay for detection of membrane changes after cryostorage. Mol Hum Reprod. 1999; 5: 109–115. doi: 10.1093/molehr/5.2.109 10065865

[72] Oosterhuis GJ, Mulder AB, Kalsbeek-Batenburg E, Lambalk CB, Schoemaker J, Vermes I. Measuring apoptosis in human spermatozoa: a biological assay for semen quality? Fertil Steril. 2000; 74: 245–250. doi: 10.1016/s0015-0282(00)00623-3 10927039

[73] Shen HM, Dai J, Chia SE, Lim A, Ong CN. Detection of apoptotic alterations in sperm in subfertile patients and their correlations with sperm quality. Hum Reprod. 2002; 17: 1266–1273. doi: 10.1093/humrep/17.5.1266 11980750

[74] Zhang HB, Lu SM, Ma CY, Wang L, Li X, Chen ZJ. Early apoptotic changes in human spermatozoa and their relationships with conventional semen parameters and sperm DNA fragmentation. Asian J Androl. 2008; 10: 227–235. doi: 10.1111/j.1745-7262.2008.00295.x 18097533

[75] Gandini L, Lombardo F, Paoli D, Caponecchia L, Familiari G, Verlengia C, et al. Study of apoptotic DNA fragmentation in human spermatozoa. Hum Reprod. 2000; 15: 830–839. doi: 10.1093/humrep/15.4.830 10739828

[76] Ammar O, Mehdi M, Tekeya O, Neffati F, Haouas Z. Novel association between apoptotic sperm biomarkers with seminal biochemical parameters and acetylcholinesterase activity in patients with teratozoospermia. J Assist Reprod Genet. 2019; 36: 2367–2378. doi: 10.1007/s10815-019-01579-7 31512048 PMC6885487

[77] Agarwal A, Saleh RA. Role of oxidants in male infertility: rationale, significance, and treatment. Urol Clin North Am. 2002; 29: 817–827. doi: 10.1016/s0094-0143(02)00081-2 12516754

[78] Aitken RJ, Harkiss D, Buckingham DW. Analysis of lipid peroxidation mechanisms in human spermatozoa. Mol. Reprod. Dev. 1993; 35: 302–315. doi: 10.1002/mrd.1080350313 8352936

